# Consultations

**Published:** 1908-07-11

**Authors:** W. H. Maidlow


### CONSULTATIONS.

By W. H. Maidlow, M.D., F.R.C.S.

In general practice these procedures are divisible into those essentially necessary and those expedient, and each kind may depend on the wish of the doctor or that of the patient. Putting aside quibbling about the significance of the word "essential," our thoughts in this division are concerned with the patient's welfare entirely. Thus there may be doubt about diagnosis, prognosis, or treatment: honest doubt whether we are doing the best possible.

On the one hand we are not to put the patient to needless expense; on the other hand, within human limits, we are to spare no effort. We are supposed to know, although we realise our frailty and the limits of our knowledge, whether anyone else can help. We may put the case to the patient: sometimes in advising a consultation; sometimes in leaving it to him to decide. In the second group there are several reasons. We may hope for a renewal or return of confidence; or to share responsibility in a grave or important case: we may wish to have an error explained, concealed, or "antidoted"; the patient may desire a consultation for his or her satisfaction on general grounds. In any of these cases we ought to fall in with the desire and foresee it.

We do not regard ourselves as a corporation with its own secrets and having a right to work entirely for its own advantage. We have a position of trust, and we are to be frank with those who trust us. The desire to conceal and recover from error is perfectly legitimate in the struggle for existence, and the thoughtless exposure of error by another rarely benefits the patient, for the latter may lose faith in his doctor, who in other respects has fully justified the conference reposed in him. Yet in no case must the patient suffer. If he has a pleural effusion diagnosed pneumonia, it must, of course be tapped if his dyspnœa cannot otherwise be relieved. In some cases the patient has sense enough to know a mistake is justifiable; then a difference of opinion may properly be explained. Only drunkenness or gross carelessness deserve no leniency. Therein our reputation as a body of honourable men is at stake.

The majority of general practitioners nowadays are painstaking: feeling we have done our best we are very ready for "counsel's opinion," and but too pleased to find our consultant less puzzled or helpless than ourselves. We ought never to fear a consultation when we remember the mistakes of our great teachers as witnessed in hospitals; when we remember what a huge subject medicine is, and its rapid advances. We witness mistakes in such an exact science as engineering, and we know of terrible miscarriages of the law. Lawyers constantly seek "counsel's opinion." Even so do we need help.

A word or two as to the management of the consultation. The consultant should wait for the consulter, who must in turn be punctual. The former follows the latter (after hearing the outline of the case and certain details which it may be best to discuss privately, yet with no air of mystery or secrecy), and is thus introduced to the patient or the friends. He should precede the latter on leaving the room and have no last word behind the latter's back. They then deliberate in another room. Then the consultant should enter first and deliver the verdict and retire first. This is not jugglery, but only due dignity and management. We but do to others as we would be done by, and the patient is always to gain. Yet we are not to harm thereby our professional brethren. Medical etiquette is apt to be derided, but it is only to be derided when badly exemplified and badly understood.

There are consultants and consultants. The ideal consultant listens carefully, starts with no preconceived ideas about the doctor or the patient, weighs the difficulties of the case, realising that a general practitioner may be as good a man as himself (even as Sir James Paget did), is gentle and kind and thoughtful to the patient, careful to make no needless investigations, and finally delivers his verdict as the result of the deliberation of two heads. In the matter of fees dichotomy is objectionable. The usual attendant is to be satisfied with a reasonable fee for his assistance to the consultant and the time or trouble he has taken. He is to find out the consultant's fee and to see that it is obtained.

The consultant should in no case see such a patient thus introduced to him without the wish of the introducer, and he is not to take on that patient as a regular attendant for that illness. In this matter there is often experienced the greatest difficulty in deciding how one is to act for the patient's greatest benefit. On the whole the best plan seems to be to wait for the patient to express his wishes to the usual attendant, who, if he be wise, will gracefully retire should his patient prefer the continued attendance of the consultant. That is a method recommended by practitioners of experience, and it seems the proper line to adopt. Really it is all a matter of tact. Tact is educated sensibility.

